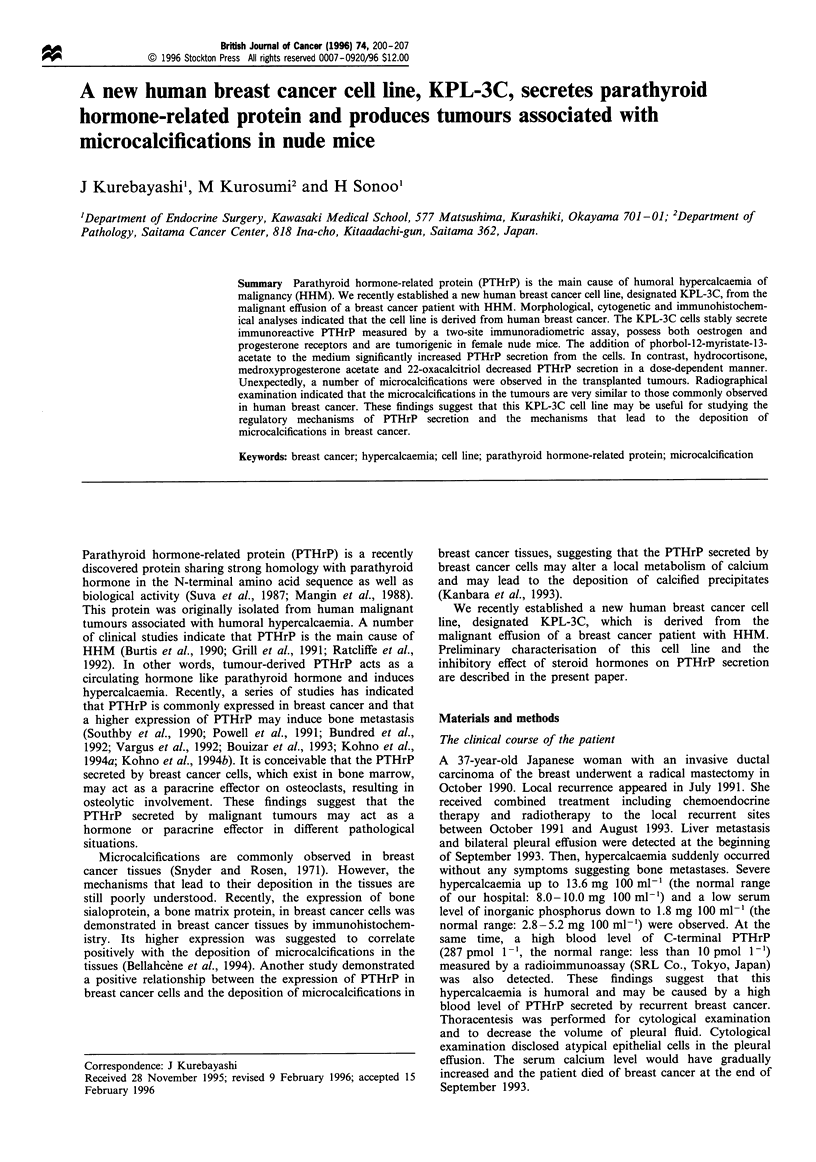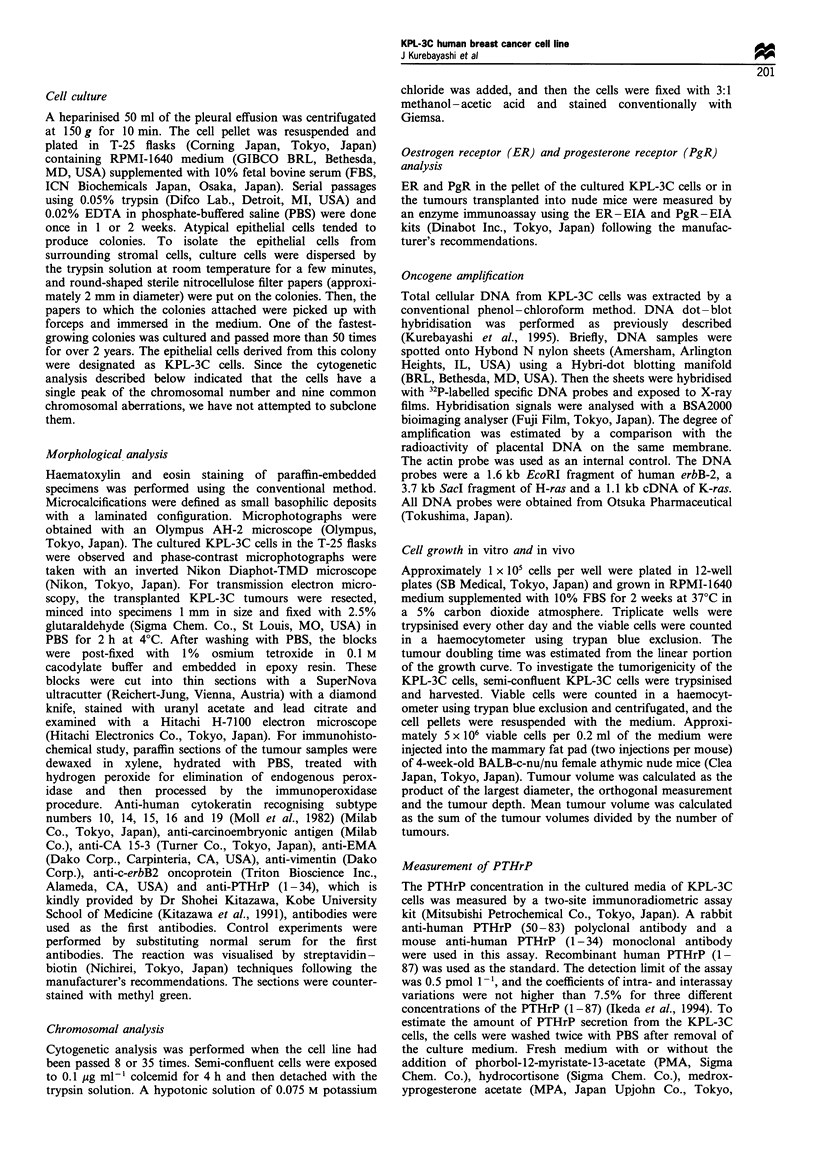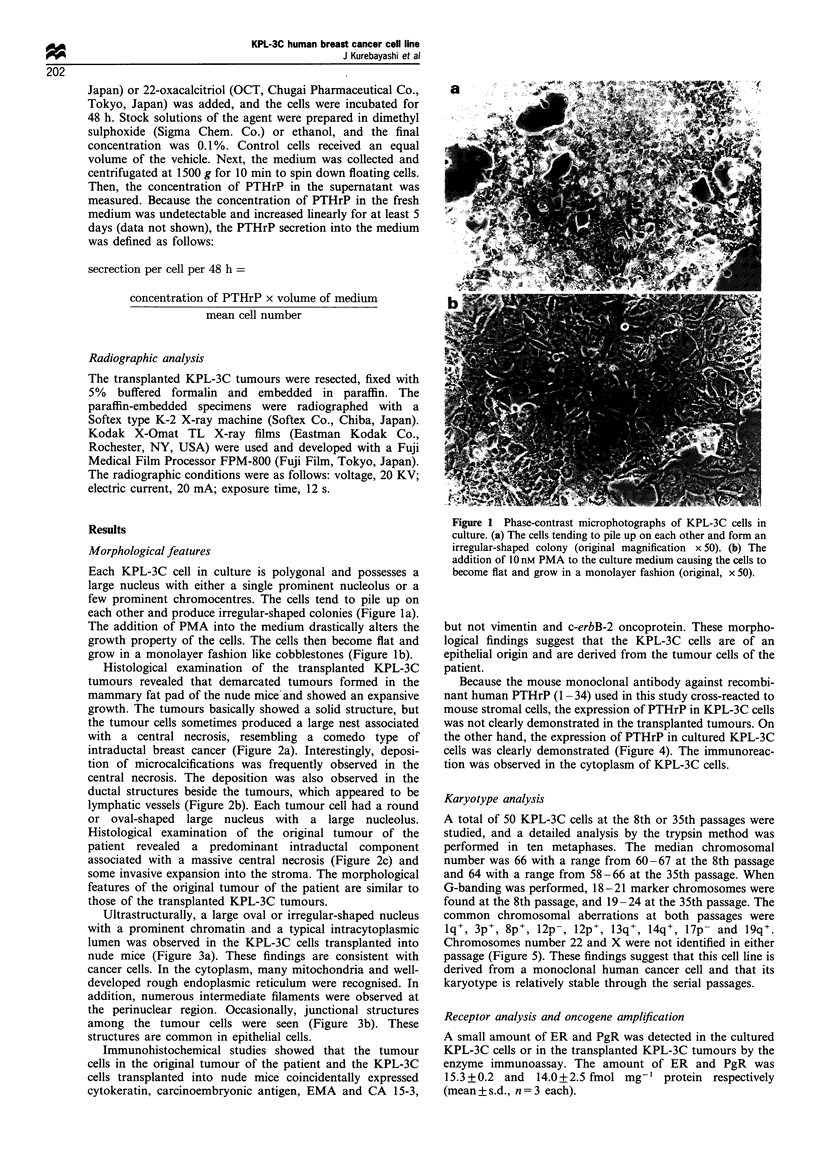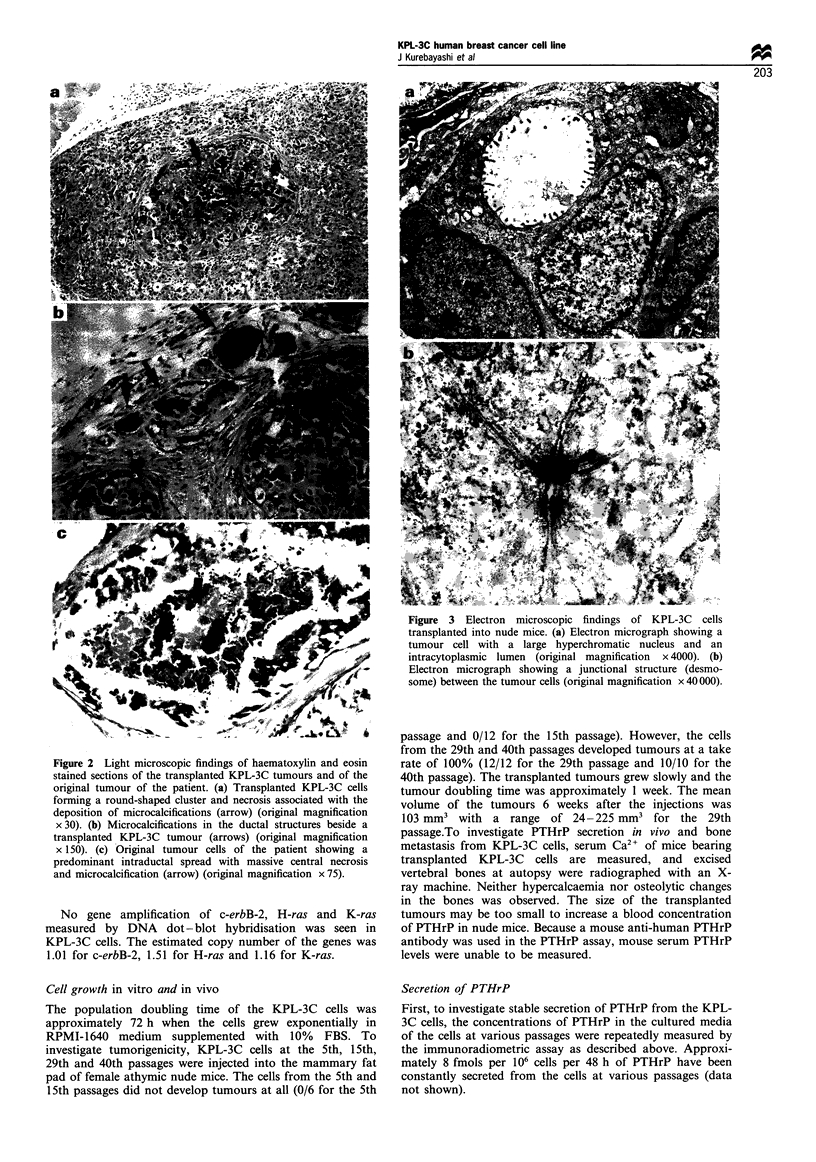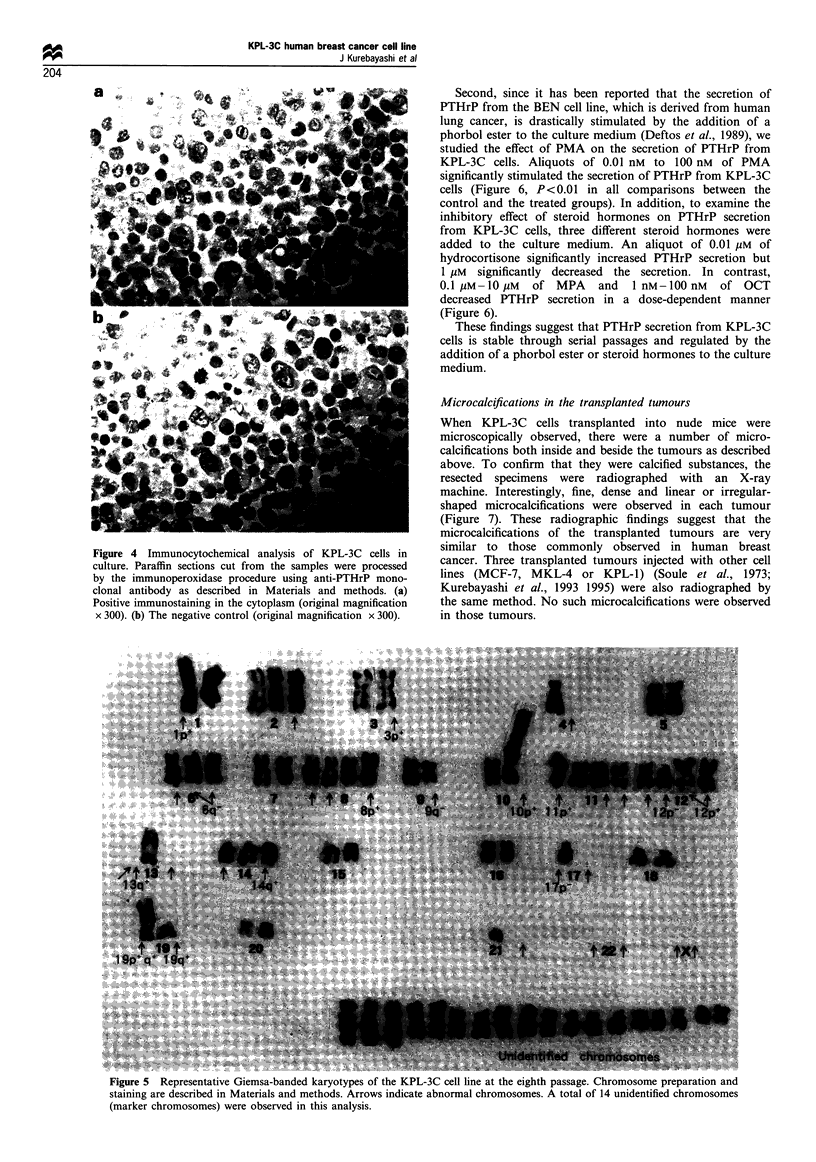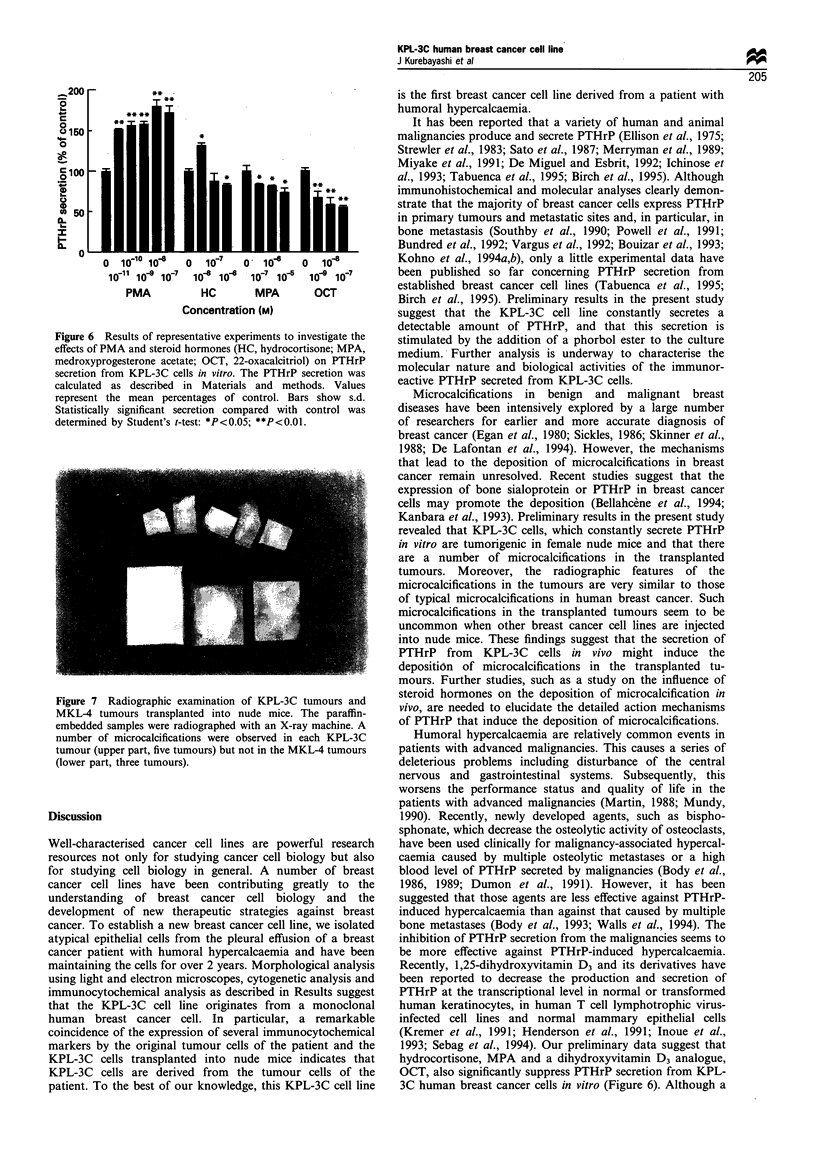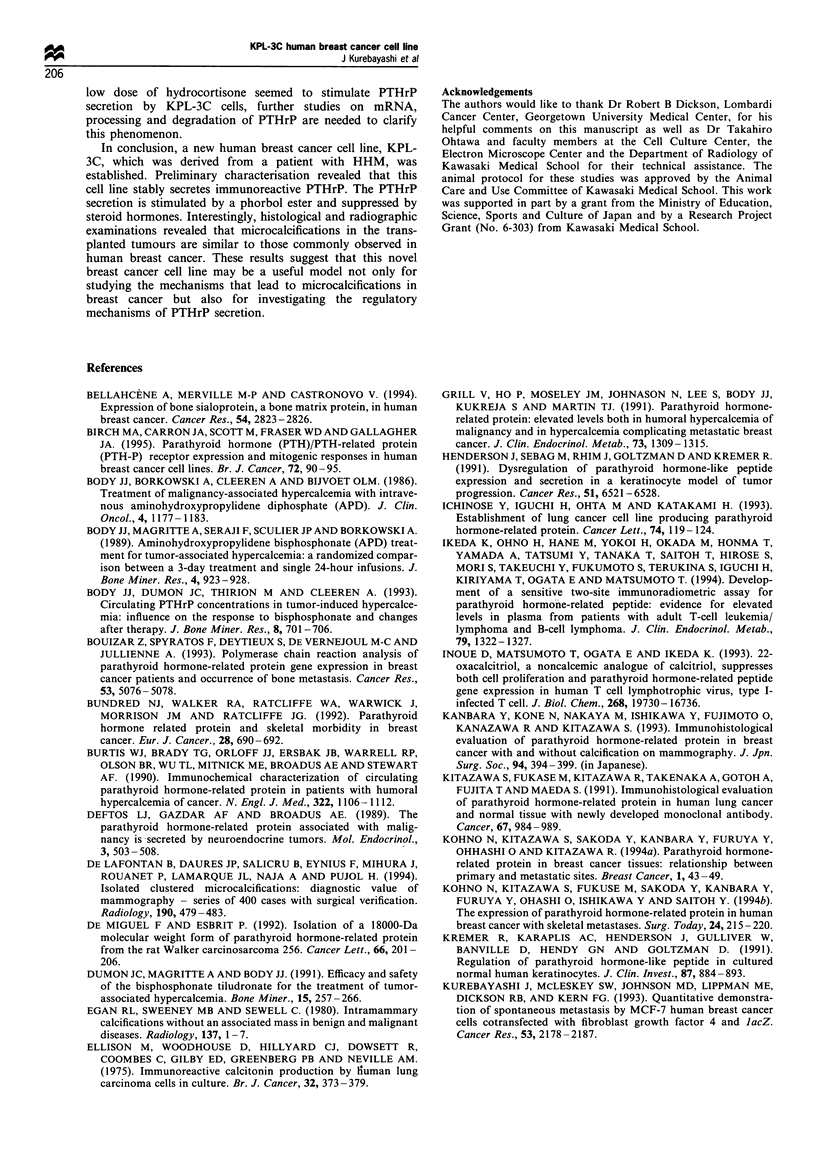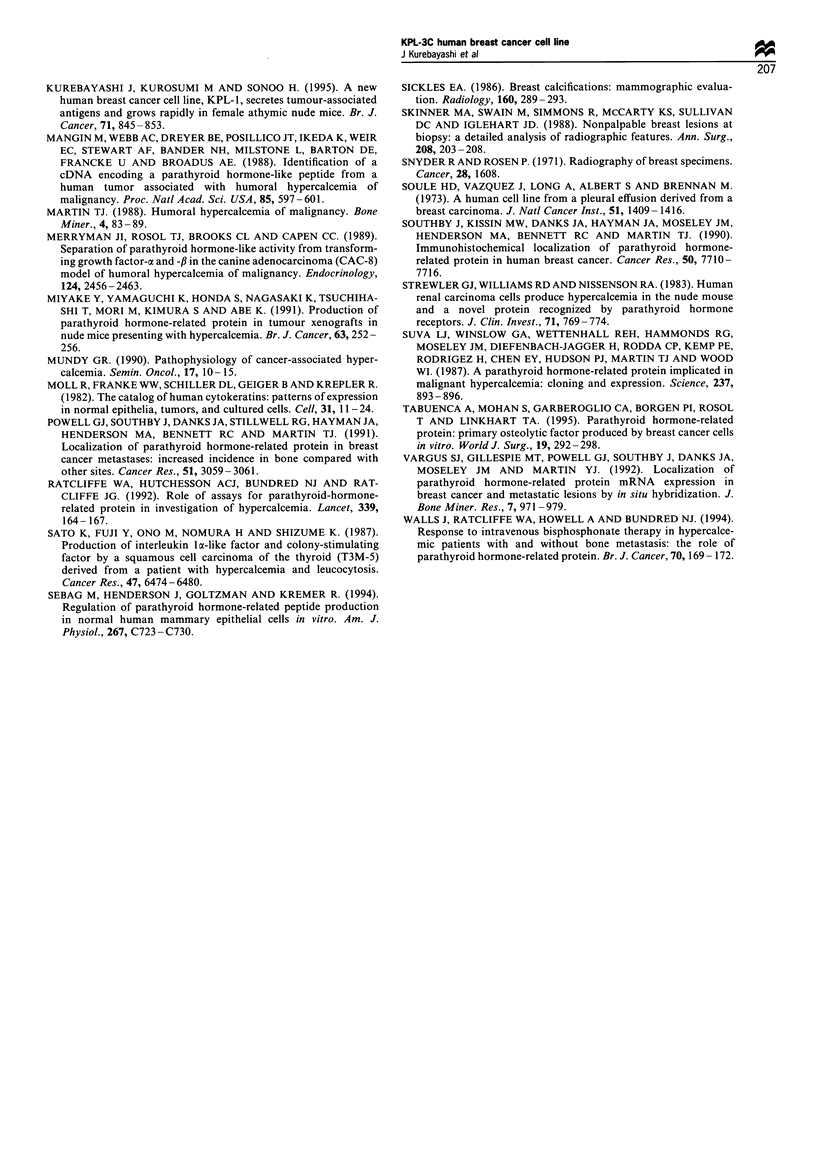# A new human breast cancer cell line, KPL-3C, secretes parathyroid hormone-related protein and produces tumours associated with microcalcifications in nude mice.

**DOI:** 10.1038/bjc.1996.338

**Published:** 1996-07

**Authors:** J. Kurebayashi, M. Kurosumi, H. Sonoo

**Affiliations:** Department of Endocrine Surgery, Kawasaki Medical School, Okayama, Japan.

## Abstract

**Images:**


British Journal of Cancer (1996) 74, 200-207
© 1996 Stockton Press All rights reserved 0007-0920/96 $12.00

### A new human breast cancer cell line, KPL-3C, secretes parathyroid hormone-related protein and produces tumours associated with microcalcifications in nude mice

J Kurebayashi[1], M Kurosumi[2] and H Sonoo[1]

[1]*Department of Endocrine Surgery, Kawasaki Medical School, 577 Matsushima, Kurashiki, Okayama 701-01;* [2]*Department of Pathology, Saitama Cancer Center, 818 Ina-cho, Kitaadachi-gun, Saitama 362, Japan.*

**Summary** Parathyroid hormone-related protein (PTHrP) is the main cause of humoral hypercalcaemia of malignancy (HHM). We recently established a new human breast cancer cell line, designated KPL-3C, from the malignant effusion of a breast cancer patient with HHM. Morphological, cytogenetic and immunohistochemical analyses indicated that the cell line is derived from human breast cancer. The KPL-3C cells stably secrete immunoreactive PTHrP measured by a two-site immunoradiometric assay, possess both oestrogen and progesterone receptors and are tumorigenic in female nude mice. The addition of phorbol-12-myristate-13-acetate to the medium significantly increased PTHrP secretion from the cells. In contrast, hydrocortisone, medroxyprogesterone acetate and 22-oxacalcitriol decreased PTHrP secretion in a dose-dependent manner. Unexpectedly, a number of microcalcifications were observed in the transplanted tumours. Radiographical examination indicated that the microcalcifications in the tumours are very similar to those commonly observed in human breast cancer. These findings suggest that this KPL-3C cell line may be useful for studying the regulatory mechanisms of PTHrP secretion and the mechanisms that lead to the deposition of microcalcifications in breast cancer.

**Keywords:** breast cancer; hypercalcaemia; cell line; parathyroid hormone-related protein; microcalcification

Parathyroid hormone-related protein (PTHrP) is a recently discovered protein sharing strong homology with parathyroid hormone in the N-terminal amino acid sequence as well as biological activity (Suva *et al.*, 1987; Mangin *et al.*, 1988). This protein was originally isolated from human malignant tumours associated with humoral hypercalcaemia. A number of clinical studies indicate that PTHrP is the main cause of HHM (Burtis *et al.*, 1990; Grill *et al.*, 1991; Ratcliffe *et al.*, 1992). In other words, tumour-derived PTHrP acts as a circulating hormone like parathyroid hormone and induces hypercalcaemia. Recently, a series of studies has indicated that PTHrP is commonly expressed in breast cancer and that a higher expression of PTHrP may induce bone metastasis (Southby *et al.*, 1990; Powell *et al.*, 1991; Bundred *et al.*, 1992; Vargus *et al.*, 1992; Bouizar *et al.*, 1993; Kohno *et al.*, 1994*a*; Kohno *et al.*, 1994*b*). It is conceivable that the PTHrP secreted by breast cancer cells, which exist in bone marrow, may act as a paracrine effector on osteoclasts, resulting in osteolytic involvement. These findings suggest that the PTHrP secreted by malignant tumours may act as a hormone or paracrine effector in different pathological situations.

Microcalcifications are commonly observed in breast cancer tissues (Snyder and Rosen, 1971). However, the mechanisms that lead to their deposition in the tissues are still poorly understood. Recently, the expression of bone sialoprotein, a bone matrix protein, in breast cancer cells was demonstrated in breast cancer tissues by immunohistochemistry. Its higher expression was suggested to correlate positively with the deposition of microcalcifications in the tissues (Bellahcène *et al.*, 1994). Another study demonstrated a positive relationship between the expression of PTHrP in breast cancer cells and the deposition of microcalcifications in breast cancer tissues, suggesting that the PTHrP secreted by breast cancer cells may alter a local metabolism of calcium and may lead to the deposition of calcified precipitates (Kanbara *et al.*, 1993).

We recently established a new human breast cancer cell line, designated KPL-3C, which is derived from the malignant effusion of a breast cancer patient with HHM. Preliminary characterisation of this cell line and the inhibitory effect of steroid hormones on PTHrP secretion are described in the present paper.

### Materials and methods

### *The clinical course of the patient*

A 37-year-old Japanese woman with an invasive ductal carcinoma of the breast underwent a radical mastectomy in October 1990. Local recurrence appeared in July 1991. She received combined treatment including chemoendocrine therapy and radiotherapy to the local recurrent sites between October 1991 and August 1993. Liver metastasis and bilateral pleural effusion were detected at the beginning of September 1993. Then, hypercalcaemia suddenly occurred without any symptoms suggesting bone metastases. Severe hypercalcaemia up to 13.6 mg 100 $ml^{-1}$ (the normal range of our hospital: 8.0-10.0 mg 100 $ml^{-1}$) and a low serum level of inorganic phosphorus down to 1.8 mg 100 $ml^{-1}$ (the normal range: 2.8-5.2 mg 100 $ml^{-1}$) were observed. At the same time, a high blood level of C-terminal PTHrP (287 pmol $l^{-1}$, the normal range: less than 10 pmol $l^{-1}$) measured by a radioimmunoassay (SRL Co., Tokyo, Japan) was also detected. These findings suggest that this hypercalcaemia is humoral and may be caused by a high blood level of PTHrP secreted by recurrent breast cancer. Thoracentesis was performed for cytological examination and to decrease the volume of pleural fluid. Cytological examination disclosed atypical epithelial cells in the pleural effusion. The serum calcium level would have gradually increased and the patient died of breast cancer at the end of September 1993.

Correspondence: J Kurebayashi
Received 28 November 1995; revised 9 February 1996; accepted 15 February 1996

*Cell culture*

A heparinised 50 ml of the pleural effusion was centrifugated at 150 *g* for 10 min. The cell pellet was resuspended and plated in T-25 flasks (Corning Japan, Tokyo, Japan) containing RPMI-1640 medium (GIBCO BRL, Bethesda, MD, USA) supplemented with 10% fetal bovine serum (FBS, ICN Biochemicals Japan, Osaka, Japan). Serial passages using 0.05% trypsin (Difco Lab., Detroit, MI, USA) and 0.02% EDTA in phosphate-buffered saline (PBS) were done once in 1 or 2 weeks. Atypical epithelial cells tended to produce colonies. To isolate the epithelial cells from surrounding stromal cells, culture cells were dispersed by the trypsin solution at room temperature for a few minutes, and round-shaped sterile nitrocellulose filter papers (approximately 2 mm in diameter) were put on the colonies. Then, the papers to which the colonies attached were picked up with forceps and immersed in the medium. One of the fastest-growing colonies was cultured and passed more than 50 times for over 2 years. The epithelial cells derived from this colony were designated as KPL-3C cells. Since the cytogenetic analysis described below indicated that the cells have a single peak of the chromosomal number and nine common chromosomal aberrations, we have not attempted to subclone them.

*Morphological analysis*

Haematoxylin and eosin staining of paraffin-embedded specimens was performed using the conventional method. Microcalcifications were defined as small basophilic deposits with a laminated configuration. Microphotographs were obtained with an Olympus AH-2 microscope (Olympus, Tokyo, Japan). The cultured KPL-3C cells in the T-25 flasks were observed and phase-contrast microphotographs were taken with an inverted Nikon Diaphot-TMD microscope (Nikon, Tokyo, Japan). For transmission electron microscopy, the transplanted KPL-3C tumours were resected, minced into specimens 1 mm in size and fixed with 2.5% glutaraldehyde (Sigma Chem. Co., St Louis, MO, USA) in PBS for 2 h at 4°C. After washing with PBS, the blocks were post-fixed with 1% osmium tetroxide in 0.1 M cacodylate buffer and embedded in epoxy resin. These blocks were cut into thin sections with a SuperNova ultracutter (Reichert-Jung, Vienna, Austria) with a diamond knife, stained with uranyl acetate and lead citrate and examined with a Hitachi H-7100 electron microscope (Hitachi Electronics Co., Tokyo, Japan). For immunohistochemical study, paraffin sections of the tumour samples were dewaxed in xylene, hydrated with PBS, treated with hydrogen peroxide for elimination of endogenous peroxidase and then processed by the immunoperoxidase procedure. Anti-human cytokeratin recognising subtype numbers 10, 14, 15, 16 and 19 (Moll *et al.*, 1982) (Milab Co., Tokyo, Japan), anti-carcinoembryonic antigen (Milab Co.), anti-CA 15-3 (Turner Co., Tokyo, Japan), anti-EMA (Dako Corp., Carpinteria, CA, USA), anti-vimentin (Dako Corp.), anti-c-*erb*B2 oncoprotein (Triton Bioscience Inc., Alameda, CA, USA) and anti-PTHrP (1–34), which is kindly provided by Dr Shohei Kitazawa, Kobe University School of Medicine (Kitazawa *et al.*, 1991), antibodies were used as the first antibodies. Control experiments were performed by substituting normal serum for the first antibodies. The reaction was visualised by streptavidin–biotin (Nichirei, Tokyo, Japan) techniques following the manufacturer's recommendations. The sections were counter-stained with methyl green.

*Chromosomal analysis*

Cytogenetic analysis was performed when the cell line had been passed 8 or 35 times. Semi-confluent cells were exposed to 0.1 $\mu$g ml$^{-1}$ colcemid for 4 h and then detached with the trypsin solution. A hypotonic solution of 0.075 M potassium chloride was added, and then the cells were fixed with 3:1 methanol–acetic acid and stained conventionally with Giemsa.

*Oestrogen receptor (ER) and progesterone receptor (PgR) analysis*

ER and PgR in the pellet of the cultured KPL-3C cells or in the tumours transplanted into nude mice were measured by an enzyme immunoassay using the ER–EIA and PgR–EIA kits (Dinabot Inc., Tokyo, Japan) following the manufacturer's recommendations.

*Oncogene amplification*

Total cellular DNA from KPL-3C cells was extracted by a conventional phenol–chloroform method. DNA dot–blot hybridisation was performed as previously described (Kurebayashi *et al.*, 1995). Briefly, DNA samples were spotted onto Hybond N nylon sheets (Amersham, Arlington Heights, IL, USA) using a Hybri-dot blotting manifold (BRL, Bethesda, MD, USA). Then the sheets were hybridised with $^{32}$P-labelled specific DNA probes and exposed to X-ray films. Hybridisation signals were analysed with a BSA2000 bioimaging analyser (Fuji Film, Tokyo, Japan). The degree of amplification was estimated by a comparison with the radioactivity of placental DNA on the same membrane. The actin probe was used as an internal control. The DNA probes were a 1.6 kb *Eco*RI fragment of human *erb*B-2, a 3.7 kb *Sac*I fragment of H-*ras* and a 1.1 kb cDNA of K-*ras*. All DNA probes were obtained from Otsuka Pharmaceutical (Tokushima, Japan).

*Cell growth* in vitro *and* in vivo

Approximately $1 \times 10^5$ cells per well were plated in 12-well plates (SB Medical, Tokyo, Japan) and grown in RPMI-1640 medium supplemented with 10% FBS for 2 weeks at 37°C in a 5% carbon dioxide atmosphere. Triplicate wells were trypsinised every other day and the viable cells were counted in a haemocytometer using trypan blue exclusion. The tumour doubling time was estimated from the linear portion of the growth curve. To investigate the tumorigenicity of the KPL-3C cells, semi-confluent KPL-3C cells were trypsinised and harvested. Viable cells were counted in a haemocytometer using trypan blue exclusion and centrifugated, and the cell pellets were resuspended with the medium. Approximately $5 \times 10^6$ viable cells per 0.2 ml of the medium were injected into the mammary fat pad (two injections per mouse) of 4-week-old BALB-c-nu/nu female athymic nude mice (Clea Japan, Tokyo, Japan). Tumour volume was calculated as the product of the largest diameter, the orthogonal measurement and the tumour depth. Mean tumour volume was calculated as the sum of the tumour volumes divided by the number of tumours.

*Measurement of PTHrP*

The PTHrP concentration in the cultured media of KPL-3C cells was measured by a two-site immunoradiometric assay kit (Mitsubishi Petrochemical Co., Tokyo, Japan). A rabbit anti-human PTHrP (50–83) polyclonal antibody and a mouse anti-human PTHrP (1–34) monoclonal antibody were used in this assay. Recombinant human PTHrP (1–87) was used as the standard. The detection limit of the assay was 0.5 pmol l$^{-1}$, and the coefficients of intra- and interassay variations were not higher than 7.5% for three different concentrations of the PTHrP (1–87) (Ikeda *et al.*, 1994). To estimate the amount of PTHrP secretion from the KPL-3C cells, the cells were washed twice with PBS after removal of the culture medium. Fresh medium with or without the addition of phorbol-12-myristate-13-acetate (PMA, Sigma Chem. Co.), hydrocortisone (Sigma Chem. Co.), medroxyprogesterone acetate (MPA, Japan Upjohn Co., Tokyo,

Japan) or 22-oxacalcitriol (OCT, Chugai Pharmaceutical Co., Tokyo, Japan) was added, and the cells were incubated for 48 h. Stock solutions of the agent were prepared in dimethyl sulphoxide (Sigma Chem. Co.) or ethanol, and the final concentration was 0.1%. Control cells received an equal volume of the vehicle. Next, the medium was collected and centrifugated at 1500 $g$ for 10 min to spin down floating cells. Then, the concentration of PTHrP in the supernatant was measured. Because the concentration of PTHrP in the fresh medium was undetectable and increased linearly for at least 5 days (data not shown), the PTHrP secretion into the medium was defined as follows:

$$\text{secretion per cell per 48 h} = \frac{\text{concentration of PTHrP} \times \text{volume of medium}}{\text{mean cell number}}$$

*Radiographic analysis*

The transplanted KPL-3C tumours were resected, fixed with 5% buffered formalin and embedded in paraffin. The paraffin-embedded specimens were radiographed with a Softex type K-2 X-ray machine (Softex Co., Chiba, Japan). Kodak X-Omat TL X-ray films (Eastman Kodak Co., Rochester, NY, USA) were used and developed with a Fuji Medical Film Processor FPM-800 (Fuji Film, Tokyo, Japan). The radiographic conditions were as follows: voltage, 20 KV; electric current, 20 mA; exposure time, 12 s.

### Results

*Morphological features*

Each KPL-3C cell in culture is polygonal and possesses a large nucleus with either a single prominent nucleolus or a few prominent chromocentres. The cells tend to pile up on each other and produce irregular-shaped colonies (Figure 1a). The addition of PMA into the medium drastically alters the growth property of the cells. The cells then become flat and grow in a monolayer fashion like cobblestones (Figure 1b).

Histological examination of the transplanted KPL-3C tumours revealed that demarcated tumours formed in the mammary fat pad of the nude mice and showed an expansive growth. The tumours basically showed a solid structure, but the tumour cells sometimes produced a large nest associated with a central necrosis, resembling a comedo type of intraductal breast cancer (Figure 2a). Interestingly, deposition of microcalcifications was frequently observed in the central necrosis. The deposition was also observed in the ductal structures beside the tumours, which appeared to be lymphatic vessels (Figure 2b). Each tumour cell had a round or oval-shaped large nucleus with a large nucleolus. Histological examination of the original tumour of the patient revealed a predominant intraductal component associated with a massive central necrosis (Figure 2c) and some invasive expansion into the stroma. The morphological features of the original tumour of the patient are similar to those of the transplanted KPL-3C tumours.

Ultrastructurally, a large oval or irregular-shaped nucleus with a prominent chromatin and a typical intracytoplasmic lumen was observed in the KPL-3C cells transplanted into nude mice (Figure 3a). These findings are consistent with cancer cells. In the cytoplasm, many mitochondria and well-developed rough endoplasmic reticulum were recognised. In addition, numerous intermediate filaments were observed at the perinuclear region. Occasionally, junctional structures among the tumour cells were seen (Figure 3b). These structures are common in epithelial cells.

Immunohistochemical studies showed that the tumour cells in the original tumour of the patient and the KPL-3C cells transplanted into nude mice coincidentally expressed cytokeratin, carcinoembryonic antigen, EMA and CA 15-3,

**Figure 1** Phase-contrast microphotographs of KPL-3C cells in culture. (**a**) The cells tending to pile up on each other and form an irregular-shaped colony (original magnification ×50). (**b**) The addition of 10 nM PMA to the culture medium causing the cells to become flat and grow in a monolayer fashion (original, ×50).

but not vimentin and c-*erb*B-2 oncoprotein. These morphological findings suggest that the KPL-3C cells are of an epithelial origin and are derived from the tumour cells of the patient.

Because the mouse monoclonal antibody against recombinant human PTHrP (1–34) used in this study cross-reacted to mouse stromal cells, the expression of PTHrP in KPL-3C cells was not clearly demonstrated in the transplanted tumours. On the other hand, the expression of PTHrP in cultured KPL-3C cells was clearly demonstrated (Figure 4). The immunoreaction was observed in the cytoplasm of KPL-3C cells.

*Karyotype analysis*

A total of 50 KPL-3C cells at the 8th or 35th passages were studied, and a detailed analysis by the trypsin method was performed in ten metaphases. The median chromosomal number was 66 with a range from 60–67 at the 8th passage and 64 with a range from 58–66 at the 35th passage. When G-banding was performed, 18–21 marker chromosomes were found at the 8th passage, and 19–24 at the 35th passage. The common chromosomal aberrations at both passages were $1q^+$, $3p^+$, $8p^+$, $12p^-$, $12p^+$, $13q^+$, $14q^+$, $17p^-$ and $19q^+$. Chromosomes number 22 and X were not identified in either passage (Figure 5). These findings suggest that this cell line is derived from a monoclonal human cancer cell and that its karyotype is relatively stable through the serial passages.

*Receptor analysis and oncogene amplification*

A small amount of ER and PgR was detected in the cultured KPL-3C cells or in the transplanted KPL-3C tumours by the enzyme immunoassay. The amount of ER and PgR was $15.3 \pm 0.2$ and $14.0 \pm 2.5$ fmol $mg^{-1}$ protein respectively (mean ± s.d., $n = 3$ each).

**Figure 2** Light microscopic findings of haematoxylin and eosin stained sections of the transplanted KPL-3C tumours and of the original tumour of the patient. (**a**) Transplanted KPL-3C cells forming a round-shaped cluster and necrosis associated with the deposition of microcalcifications (arrow) (original magnification ×30). (**b**) Microcalcifications in the ductal structures beside a transplanted KPL-3C tumour (arrows) (original magnification ×150). (**c**) Original tumour cells of the patient showing a predominant intraductal spread with massive central necrosis and microcalcification (arrow) (original magnification ×75).

No gene amplification of c-*erb*B-2, H-*ras* and K-*ras* measured by DNA dot–blot hybridisation was seen in KPL-3C cells. The estimated copy number of the genes was 1.01 for c-*erb*B-2, 1.51 for H-*ras* and 1.16 for K-*ras*.

### *Cell growth* in vitro *and* in vivo

The population doubling time of the KPL-3C cells was approximately 72 h when the cells grew exponentially in RPMI-1640 medium supplemented with 10% FBS. To investigate tumorigenicity, KPL-3C cells at the 5th, 15th, 29th and 40th passages were injected into the mammary fat pad of female athymic nude mice. The cells from the 5th and 15th passages did not develop tumours at all (0/6 for the 5th passage and 0/12 for the 15th passage). However, the cells from the 29th and 40th passages developed tumours at a take rate of 100% (12/12 for the 29th passage and 10/10 for the 40th passage). The transplanted tumours grew slowly and the tumour doubling time was approximately 1 week. The mean volume of the tumours 6 weeks after the injections was 103 mm$^3$ with a range of 24–225 mm$^3$ for the 29th passage.To investigate PTHrP secretion *in vivo* and bone metastasis from KPL-3C cells, serum $Ca^{2+}$ of mice bearing transplanted KPL-3C cells are measured, and excised vertebral bones at autopsy were radiographed with an X-ray machine. Neither hypercalcaemia nor osteolytic changes in the bones was observed. The size of the transplanted tumours may be too small to increase a blood concentration of PTHrP in nude mice. Because a mouse anti-human PTHrP antibody was used in the PTHrP assay, mouse serum PTHrP levels were unable to be measured.

**Figure 3** Electron microscopic findings of KPL-3C cells transplanted into nude mice. (**a**) Electron micrograph showing a tumour cell with a large hyperchromatic nucleus and an intracytoplasmic lumen (original magnification ×4000). (**b**) Electron micrograph showing a junctional structure (desmosome) between the tumour cells (original magnification ×40 000).

### *Secretion of PTHrP*

First, to investigate stable secretion of PTHrP from the KPL-3C cells, the concentrations of PTHrP in the cultured media of the cells at various passages were repeatedly measured by the immunoradiometric assay as described above. Approximately 8 fmols per $10^6$ cells per 48 h of PTHrP have been constantly secreted from the cells at various passages (data not shown).

**Figure 4** Immunocytochemical analysis of KPL-3C cells in culture. Paraffin sections cut from the samples were processed by the immunoperoxidase procedure using anti-PTHrP monoclonal antibody as described in Materials and methods. (**a**) Positive immunostaining in the cytoplasm (original magnification ×300). (**b**) The negative control (original magnification ×300).

Second, since it has been reported that the secretion of PTHrP from the BEN cell line, which is derived from human lung cancer, is drastically stimulated by the addition of a phorbol ester to the culture medium (Deftos *et al.*, 1989), we studied the effect of PMA on the secretion of PTHrP from KPL-3C cells. Aliquots of 0.01 nM to 100 nM of PMA significantly stimulated the secretion of PTHrP from KPL-3C cells (Figure 6, $P<0.01$ in all comparisons between the control and the treated groups). In addition, to examine the inhibitory effect of steroid hormones on PTHrP secretion from KPL-3C cells, three different steroid hormones were added to the culture medium. An aliquot of 0.01 $\mu$M of hydrocortisone significantly increased PTHrP secretion but 1 $\mu$M significantly decreased the secretion. In contrast, 0.1 $\mu$M – 10 $\mu$M of MPA and 1 nM – 100 nM of OCT decreased PTHrP secretion in a dose-dependent manner (Figure 6).

These findings suggest that PTHrP secretion from KPL-3C cells is stable through serial passages and regulated by the addition of a phorbol ester or steroid hormones to the culture medium.

*Microcalcifications in the transplanted tumours*

When KPL-3C cells transplanted into nude mice were microscopically observed, there were a number of microcalcifications both inside and beside the tumours as described above. To confirm that they were calcified substances, the resected specimens were radiographed with an X-ray machine. Interestingly, fine, dense and linear or irregular-shaped microcalcifications were observed in each tumour (Figure 7). These radiographic findings suggest that the microcalcifications of the transplanted tumours are very similar to those commonly observed in human breast cancer. Three transplanted tumours injected with other cell lines (MCF-7, MKL-4 or KPL-1) (Soule *et al.*, 1973; Kurebayashi *et al.*, 1993 1995) were also radiographed by the same method. No such microcalcifications were observed in those tumours.

**Figure 5** Representative Giemsa-banded karyotypes of the KPL-3C cell line at the eighth passage. Chromosome preparation and staining are described in Materials and methods. Arrows indicate abnormal chromosomes. A total of 14 unidentified chromosomes (marker chromosomes) were observed in this analysis.

**Figure 6** Results of representative experiments to investigate the effects of PMA and steroid hormones (HC, hydrocortisone; MPA, medroxyprogesterone acetate; OCT, 22-oxacalcitriol) on PTHrP secretion from KPL-3C cells *in vitro*. The PTHrP secretion was calculated as described in Materials and methods. Values represent the mean percentages of control. Bars show s.d. Statistically significant secretion compared with control was determined by Student's *t*-test: $*P<0.05$; $**P<0.01$.

**Figure 7** Radiographic examination of KPL-3C tumours and MKL-4 tumours transplanted into nude mice. The paraffin-embedded samples were radiographed with an X-ray machine. A number of microcalcifications were observed in each KPL-3C tumour (upper part, five tumours) but not in the MKL-4 tumours (lower part, three tumours).

### Discussion

Well-characterised cancer cell lines are powerful research resources not only for studying cancer cell biology but also for studying cell biology in general. A number of breast cancer cell lines have been contributing greatly to the understanding of breast cancer cell biology and the development of new therapeutic strategies against breast cancer. To establish a new breast cancer cell line, we isolated atypical epithelial cells from the pleural effusion of a breast cancer patient with humoral hypercalcaemia and have been maintaining the cells for over 2 years. Morphological analysis using light and electron microscopes, cytogenetic analysis and immunocytochemical analysis as described in Results suggest that the KPL-3C cell line originates from a monoclonal human breast cancer cell. In particular, a remarkable coincidence of the expression of several immunocytochemical markers by the original tumour cells of the patient and the KPL-3C cells transplanted into nude mice indicates that KPL-3C cells are derived from the tumour cells of the patient. To the best of our knowledge, this KPL-3C cell line is the first breast cancer cell line derived from a patient with humoral hypercalcaemia.

It has been reported that a variety of human and animal malignancies produce and secrete PTHrP (Ellison *et al.*, 1975; Strewler *et al.*, 1983; Sato *et al.*, 1987; Merryman *et al.*, 1989; Miyake *et al.*, 1991; De Miguel and Esbrit, 1992; Ichinose *et al.*, 1993; Tabuenca *et al.*, 1995; Birch *et al.*, 1995). Although immunohistochemical and molecular analyses clearly demonstrate that the majority of breast cancer cells express PTHrP in primary tumours and metastatic sites and, in particular, in bone metastasis (Southby *et al.*, 1990; Powell *et al.*, 1991; Bundred *et al.*, 1992; Vargus *et al.*, 1992; Bouizar *et al.*, 1993; Kohno *et al.*, 1994*a*,*b*), only a little experimental data have been published so far concerning PTHrP secretion from established breast cancer cell lines (Tabuenca *et al.*, 1995; Birch *et al.*, 1995). Preliminary results in the present study suggest that the KPL-3C cell line constantly secretes a detectable amount of PTHrP, and that this secretion is stimulated by the addition of a phorbol ester to the culture medium. Further analysis is underway to characterise the molecular nature and biological activities of the immunoreactive PTHrP secreted from KPL-3C cells.

Microcalcifications in benign and malignant breast diseases have been intensively explored by a large number of researchers for earlier and more accurate diagnosis of breast cancer (Egan *et al.*, 1980; Sickles, 1986; Skinner *et al.*, 1988; De Lafontan *et al.*, 1994). However, the mechanisms that lead to the deposition of microcalcifications in breast cancer remain unresolved. Recent studies suggest that the expression of bone sialoprotein or PTHrP in breast cancer cells may promote the deposition (Bellahcène *et al.*, 1994; Kanbara *et al.*, 1993). Preliminary results in the present study revealed that KPL-3C cells, which constantly secrete PTHrP *in vitro* are tumorigenic in female nude mice and that there are a number of microcalcifications in the transplanted tumours. Moreover, the radiographic features of the microcalcifications in the tumours are very similar to those of typical microcalcifications in human breast cancer. Such microcalcifications in the transplanted tumours seem to be uncommon when other breast cancer cell lines are injected into nude mice. These findings suggest that the secretion of PTHrP from KPL-3C cells *in vivo* might induce the deposition of microcalcifications in the transplanted tumours. Further studies, such as a study on the influence of steroid hormones on the deposition of microcalcification *in vivo*, are needed to elucidate the detailed action mechanisms of PTHrP that induce the deposition of microcalcifications.

Humoral hypercalcaemia are relatively common events in patients with advanced malignancies. This causes a series of deleterious problems including disturbance of the central nervous and gastrointestinal systems. Subsequently, this worsens the performance status and quality of life in the patients with advanced malignancies (Martin, 1988; Mundy, 1990). Recently, newly developed agents, such as bisphosphonate, which decrease the osteolytic activity of osteoclasts, have been used clinically for malignancy-associated hypercalcaemia caused by multiple osteolytic metastases or a high blood level of PTHrP secreted by malignancies (Body *et al.*, 1986, 1989; Dumon *et al.*, 1991). However, it has been suggested that those agents are less effective against PTHrP-induced hypercalcaemia than against that caused by multiple bone metastases (Body *et al.*, 1993; Walls *et al.*, 1994). The inhibition of PTHrP secretion from the malignancies seems to be more effective against PTHrP-induced hypercalcaemia. Recently, 1,25-dihydroxyvitamin $D_3$ and its derivatives have been reported to decrease the production and secretion of PTHrP at the transcriptional level in normal or transformed human keratinocytes, in human T cell lymphotrophic virus-infected cell lines and normal mammary epithelial cells (Kremer *et al.*, 1991; Henderson *et al.*, 1991; Inoue *et al.*, 1993; Sebag *et al.*, 1994). Our preliminary data suggest that hydrocortisone, MPA and a dihydroxyvitamin $D_3$ analogue, OCT, also significantly suppress PTHrP secretion from KPL-3C human breast cancer cells *in vitro* (Figure 6). Although a low dose of hydrocortisone seemed to stimulate PTHrP secretion by KPL-3C cells, further studies on mRNA, processing and degradation of PTHrP are needed to clarify this phenomenon.

In conclusion, a new human breast cancer cell line, KPL-3C, which was derived from a patient with HHM, was established. Preliminary characterisation revealed that this cell line stably secretes immunoreactive PTHrP. The PTHrP secretion is stimulated by a phorbol ester and suppressed by steroid hormones. Interestingly, histological and radiographic examinations revealed that microcalcifications in the transplanted tumours are similar to those commonly observed in human breast cancer. These results suggest that this novel breast cancer cell line may be a useful model not only for studying the mechanisms that lead to microcalcifications in breast cancer but also for investigating the regulatory mechanisms of PTHrP secretion.

**Acknowledgements**

The authors would like to thank Dr Robert B Dickson, Lombardi Cancer Center, Georgetown University Medical Center, for his helpful comments on this manuscript as well as Dr Takahiro Ohtawa and faculty members at the Cell Culture Center, the Electron Microscope Center and the Department of Radiology of Kawasaki Medical School for their technical assistance. The animal protocol for these studies was approved by the Animal Care and Use Committee of Kawasaki Medical School. This work was supported in part by a grant from the Ministry of Education, Science, Sports and Culture of Japan and by a Research Project Grant (No. 6-303) from Kawasaki Medical School.

### References

BELLAHCÈNE A, MERVILLE M-P AND CASTRONOVO V. (1994). Expression of bone sialoprotein, a bone matrix protein, in human breast cancer. *Cancer Res.*, **54,** 2823–2826.

BIRCH MA, CARRON JA, SCOTT M, FRASER WD AND GALLAGHER JA. (1995). Parathyroid hormone (PTH)/PTH-related protein (PTH-P) receptor expression and mitogenic responses in human breast cancer cell lines. *Br. J. Cancer*, **72,** 90–95.

BODY JJ, BORKOWSKI A, CLEEREN A AND BIJVOET OLM. (1986). Treatment of malignancy-associated hypercalcemia with intravenous aminohydroxypropylidene diphosphate (APD). *J. Clin. Oncol.*, **4,** 1177–1183.

BODY JJ, MAGRITTE A, SERAJI F, SCULIER JP AND BORKOWSKI A. (1989). Aminohydroxypropylidene bisphosphonate (APD) treatment for tumor-associated hypercalcemia: a randomized comparison between a 3-day treatment and single 24-hour infusions. *J. Bone Miner. Res.*, **4,** 923–928.

BODY JJ, DUMON JC, THIRION M AND CLEEREN A. (1993). Circulating PTHrP concentrations in tumor-induced hypercalcemia: influence on the response to bisphosphonate and changes after therapy. *J. Bone Miner. Res.*, **8,** 701–706.

BOUIZAR Z, SPYRATOS F, DEYTIEUX S, DE VERNEJOUL M-C AND JULLIENNE A. (1993). Polymerase chain reaction analysis of parathyroid hormone-related protein gene expression in breast cancer patients and occurrence of bone metastasis. *Cancer Res.*, **53,** 5076–5078.

BUNDRED NJ, WALKER RA, RATCLIFFE WA, WARWICK J, MORRISON JM AND RATCLIFFE JG. (1992). Parathyroid hormone related protein and skeletal morbidity in breast cancer. *Eur. J. Cancer.*, **28,** 690–692.

BURTIS WJ, BRADY TG, ORLOFF JJ, ERSBAK JB, WARRELL RP, OLSON BR, WU TL, MITNICK ME, BROADUS AE AND STEWART AF. (1990). Immunochemical characterization of circulating parathyroid hormone-related protein in patients with humoral hypercalcemia of cancer. *N. Engl. J. Med.*, **322,** 1106–1112.

DEFTOS LJ, GAZDAR AF AND BROADUS AE. (1989). The parathyroid hormone-related protein associated with malignancy is secreted by neuroendocrine tumors. *Mol. Endocrinol.*, **3,** 503–508.

DE LAFONTAN B, DAURES JP, SALICRU B, EYNIUS F, MIHURA J, ROUANET P, LAMARQUE JL, NAJA A AND PUJOL H. (1994). Isolated clustered microcalcifications: diagnostic value of mammography – series of 400 cases with surgical verification. *Radiology*, **190,** 479–483.

DE MIGUEL F AND ESBRIT P. (1992). Isolation of a 18000-Da molecular weight form of parathyroid hormone-related protein from the rat Walker carcinosarcoma 256. *Cancer Lett.*, **66,** 201–206.

DUMON JC, MAGRITTE A AND BODY JJ. (1991). Efficacy and safety of the bisphosphonate tiludronate for the treatment of tumor-associated hypercalcemia. *Bone Miner.*, **15,** 257–266.

EGAN RL, SWEENEY MB AND SEWELL C. (1980). Intramammary calcifications without an associated mass in benign and malignant diseases. *Radiology*, **137,** 1–7.

ELLISON M, WOODHOUSE D, HILLYARD CJ, DOWSETT R, COOMBES C, GILBY ED, GREENBERG PB AND NEVILLE AM. (1975). Immunoreactive calcitonin production by human lung carcinoma cells in culture. *Br. J. Cancer*, **32,** 373–379.

GRILL V, HO P, MOSELEY JM, JOHNASON N, LEE S, BODY JJ, KUKREJA S AND MARTIN TJ. (1991). Parathyroid hormone-related protein: elevated levels both in humoral hypercalcemia of malignancy and in hypercalcemia complicating metastatic breast cancer. *J. Clin. Endocrinol. Metab.*, **73,** 1309–1315.

HENDERSON J, SEBAG M, RHIM J, GOLTZMAN D AND KREMER R. (1991). Dysregulation of parathyroid hormone-like peptide expression and secretion in a keratinocyte model of tumor progression. *Cancer Res.*, **51,** 6521–6528.

ICHINOSE Y, IGUCHI H, OHTA M AND KATAKAMI H. (1993). Establishment of lung cancer cell line producing parathyroid hormone-related protein. *Cancer Lett.*, **74,** 119–124.

IKEDA K, OHNO H, HANE M, YOKOI H, OKADA M, HONMA T, YAMADA A, TATSUMI Y, TANAKA T, SAITOH T, HIROSE S, MORI S, TAKEUCHI Y, FUKUMOTO S, TERUKINA S, IGUCHI H, KIRIYAMA T, OGATA E AND MATSUMOTO T. (1994). Development of a sensitive two-site immunoradiometric assay for parathyroid hormone-related peptide: evidence for elevated levels in plasma from patients with adult T-cell leukemia/lymphoma and B-cell lymphoma. *J. Clin. Endocrinol. Metab.*, **79,** 1322–1327.

INOUE D, MATSUMOTO T, OGATA E AND IKEDA K. (1993). 22-oxacalcitriol, a noncalcemic analogue of calcitriol, suppresses both cell proliferation and parathyroid hormone-related peptide gene expression in human T cell lymphotrophic virus, type I-infected T cell. *J. Biol. Chem.*, **268,** 19730–16736.

KANBARA Y, KONE N, NAKAYA M, ISHIKAWA Y, FUJIMOTO O, KANAZAWA R AND KITAZAWA S. (1993). Immunohistological evaluation of parathyroid hormone-related protein in breast cancer with and without calcification on mammography. *J. Jpn. Surg. Soc.*, **94,** 394–399. (in Japanese).

KITAZAWA S, FUKASE M, KITAZAWA R, TAKENAKA A, GOTOH A, FUJITA T AND MAEDA S. (1991). Immunohistological evaluation of parathyroid hormone-related protein in human lung cancer and normal tissue with newly developed monoclonal antibody. *Cancer*, **67,** 984–989.

KOHNO N, KITAZAWA S, SAKODA Y, KANBARA Y, FURUYA Y, OHHASHI O AND KITAZAWA R. (1994*a*). Parathyroid hormone-related protein in breast cancer tissues: relationship between primary and metastatic sites. *Breast Cancer*, **1,** 43–49.

KOHNO N, KITAZAWA S, FUKUSE M, SAKODA Y, KANBARA Y, FURUYA Y, OHASHI O, ISHIKAWA Y AND SAITOH Y. (1994*b*). The expression of parathyroid hormone-related protein in human breast cancer with skeletal metastases. *Surg. Today*, **24,** 215–220.

KREMER R, KARAPLIS AC, HENDERSON J, GULLIVER W, BANVILLE D, HENDY GN AND GOLTZMAN D. (1991). Regulation of parathyroid hormone-like peptide in cultured normal human keratinocytes. *J. Clin. Invest.*, **87,** 884–893.

KUREBAYASHI J, MCLESKEY SW, JOHNSON MD, LIPPMAN ME, DICKSON RB, AND KERN FG. (1993). Quantitative demonstration of spontaneous metastasis by MCF-7 human breast cancer cells cotransfected with fibroblast growth factor 4 and *lacZ*. *Cancer Res.*, **53,** 2178–2187.

KUREBAYASHI J, KUROSUMI M AND SONOO H. (1995). A new human breast cancer cell line, KPL-1, secretes tumour-associated antigens and grows rapidly in female athymic nude mice. *Br. J. Cancer*, **71**, 845–853.

MANGIN M, WEBB AC, DREYER BE, POSILLICO JT, IKEDA K, WEIR EC, STEWART AF, BANDER NH, MILSTONE L, BARTON DE, FRANCKE U AND BROADUS AE. (1988). Identification of a cDNA encoding a parathyroid hormone-like peptide from a human tumor associated with humoral hypercalcemia of malignancy. *Proc. Natl Acad. Sci. USA*, **85**, 597–601.

MARTIN TJ. (1988). Humoral hypercalcemia of malignancy. *Bone Miner.*, **4**, 83–89.

MERRYMAN JI, ROSOL TJ, BROOKS CL AND CAPEN CC. (1989). Separation of parathyroid hormone-like activity from transforming growth factor-$\alpha$ and -$\beta$ in the canine adenocarcinoma (CAC-8) model of humoral hypercalcemia of malignancy. *Endocrinology*, **124**, 2456–2463.

MIYAKE Y, YAMAGUCHI K, HONDA S, NAGASAKI K, TSUCHIHASHI T, MORI M, KIMURA S AND ABE K. (1991). Production of parathyroid hormone-related protein in tumour xenografts in nude mice presenting with hypercalcemia. *Br. J. Cancer*, **63**, 252–256.

MUNDY GR. (1990). Pathophysiology of cancer-associated hypercalcemia. *Semin. Oncol.*, **17**, 10–15.

MOLL R, FRANKE WW, SCHILLER DL, GEIGER B AND KREPLER R. (1982). The catalog of human cytokeratins: patterns of expression in normal epithelia, tumors, and cultured cells. *Cell*, **31**, 11–24.

POWELL GJ, SOUTHBY J, DANKS JA, STILLWELL RG, HAYMAN JA, HENDERSON MA, BENNETT RC AND MARTIN TJ. (1991). Localization of parathyroid hormone-related protein in breast cancer metastases: increased incidence in bone compared with other sites. *Cancer Res.*, **51**, 3059–3061.

RATCLIFFE WA, HUTCHESSON ACJ, BUNDRED NJ AND RATCLIFFE JG. (1992). Role of assays for parathyroid-hormone-related protein in investigation of hypercalcemia. *Lancet*, **339**, 164–167.

SATO K, FUJI Y, ONO M, NOMURA H AND SHIZUME K. (1987). Production of interleukin 1$\alpha$-like factor and colony-stimulating factor by a squamous cell carcinoma of the thyroid (T3M-5) derived from a patient with hypercalcemia and leucocytosis. *Cancer Res.*, **47**, 6474–6480.

SEBAG M, HENDERSON J, GOLTZMAN AND KREMER R. (1994). Regulation of parathyroid hormone-related peptide production in normal human mammary epithelial cells *in vitro*. *Am. J. Physiol.*, **267**, C723–C730.

SICKLES EA. (1986). Breast calcifications: mammographic evaluation. *Radiology*, **160**, 289–293.

SKINNER MA, SWAIN M, SIMMONS R, MCCARTY KS, SULLIVAN DC AND IGLEHART JD. (1988). Nonpalpable breast lesions at biopsy: a detailed analysis of radiographic features. *Ann. Surg.*, **208**, 203–208.

SNYDER R AND ROSEN P. (1971). Radiography of breast specimens. *Cancer*, **28**, 1608.

SOULE HD, VAZQUEZ J, LONG A, ALBERT S AND BRENNAN M. (1973). A human cell line from a pleural effusion derived from a breast carcinoma. *J. Natl Cancer Inst.*, **51**, 1409–1416.

SOUTHBY J, KISSIN MW, DANKS JA, HAYMAN JA, MOSELEY JM, HENDERSON MA, BENNETT RC AND MARTIN TJ. (1990). Immunohistochemical localization of parathyroid hormone-related protein in human breast cancer. *Cancer Res.*, **50**, 7710–7716.

STREWLER GJ, WILLIAMS RD AND NISSENSON RA. (1983). Human renal carcinoma cells produce hypercalcemia in the nude mouse and a novel protein recognized by parathyroid hormone receptors. *J. Clin. Invest.*, **71**, 769–774.

SUVA LJ, WINSLOW GA, WETTENHALL REH, HAMMONDS RG, MOSELEY JM, DIEFENBACH-JAGGER H, RODDA CP, KEMP PE, RODRIGEZ H, CHEN EY, HUDSON PJ, MARTIN TJ AND WOOD WI. (1987). A parathyroid hormone-related protein implicated in malignant hypercalcemia: cloning and expression. *Science*, **237**, 893–896.

TABUENCA A, MOHAN S, GARBEROGLIO CA, BORGEN PI, ROSOL T AND LINKHART TA. (1995). Parathyroid hormone-related protein: primary osteolytic factor produced by breast cancer cells *in vitro*. *World J. Surg.*, **19**, 292–298.

VARGUS SJ, GILLESPIE MT, POWELL GJ, SOUTHBY J, DANKS JA, MOSELEY JM AND MARTIN YJ. (1992). Localization of parathyroid hormone-related protein mRNA expression in breast cancer and metastatic lesions by *in situ* hybridization. *J. Bone Miner. Res.*, **7**, 971–979.

WALLS J, RATCLIFFE WA, HOWELL A AND BUNDRED NJ. (1994). Response to intravenous bisphosphonate therapy in hypercalcemic patients with and without bone metastasis: the role of parathyroid hormone-related protein. *Br. J. Cancer*, **70**, 169–172.